# Mechanical and Thermal Characterization of Styrenic Thermoplastic Elastomer Compounds with Recycled Content for Sustainable Automotive Applications

**DOI:** 10.3390/polym18131646

**Published:** 2026-07-02

**Authors:** Flavia Cano, Matilde Arese, Graziano Brocani, Silvia Ponti, Gabriele Ciaccio, Valentina Brunella

**Affiliations:** 1 NIS Interdepartmental Centre, Department of Chemistry, University of Turin, Via P. Giuria 7, 10125 Turin, Italy; flavia.cano@unito.it (F.C.); matilde.arese@unito.it (M.A.); 2 Stellantis, C.so Agnelli 220, 10135 Turin, Italy; graziano.brocani@crf.it (G.B.); silvia.ponti@crf.it (S.P.); gabriele.ciaccio@crf.it (G.C.)

**Keywords:** TPS, SEPS/PP blends, recycling, mechanical performance, oil migration, sustainability

## Abstract

In the context of increasing environmental awareness and the transition toward a circular material economy, the development of sustainable polymeric materials has become a key focus of industrial research. Within this framework, thermoplastic elastomers (TPEs) represent a promising class of materials that combine the elasticity of rubbers with the processability and recyclability of thermoplastics. Their ability to incorporate recycled content further enhances their potential for reducing environmental impact in advanced automotive applications. This study investigates styrenic thermoplastic elastomers (TPS) based on a SEPS (Styrene–Ethylene–Propylene–Styrene) and polypropylene matrix containing over 50% recycled content, with the aim of evaluating the influence of recycled material on structure and performance. TGA, DSC, and ATR-FTIR analyses revealed comparable degradation behavior and similar chemical features between virgin and recycled compounds, while minor differences were possibly related to variations in the plasticizer fraction and polymer-oil interactions. These differences did not significantly compromise the mechanical integrity of the recycled materials under the conditions investigated. Mechanical tests (tensile, tear, hardness, compression set) confirmed that recycled TPS maintains mechanical performance comparable to virgin formulations, while accelerated weathering resulted in minimal color variation and excellent surface appearance retention. Overall, TPS with high recycled content exhibit stable thermal, chemical, and mechanical behavior, confirming their suitability as sustainable alternatives for automotive components.

## 1. Introduction

The automotive sector is currently undergoing a major transformation, driven by increasingly stringent environmental regulations, ambitious decarbonization targets, and the rising demand for sustainable mobility solutions [1,2,3]. One of the central strategies in this transition is the replacement of conventional materials with lightweight polymers, which reduce vehicle mass and, consequently, fuel consumption and greenhouse gas emissions [4,5]. Within this broader class of polymers, elastomeric materials play a particularly critical role. Elastomers are everywhere in vehicles, being used in weatherstrips, gaskets, hoses, vibration-damping components, and interior elements [6,7]. Traditionally, these parts have been manufactured from thermoset rubbers, which are chemically crosslinked and therefore non-reprocessable at the end of their service life. This inherent limitation significantly complicates recycling and contributes to the accumulation of non-recovered waste from end-of-life vehicles (ELVs) [8,9].

Addressing this issue requires not only material innovation but also more effective recycling strategies. At present, the recycling of polymers, including elastomers, follows two principal pathways: mechanical and chemical recycling. While chemical recycling holds long-term promise, its high energy demand has so far restricted large-scale deployment. In contrast, mechanical recycling offers a more immediate and resource-efficient route for establishing closed-loop systems [10,11]. However, the quality of recycled material strongly depends on the origin of the waste. Post-industrial recycled (PIR) streams, generated during manufacturing, are typically clean and homogeneous, producing high-grade recycled material with minimal contamination [12,13]. Post-consumer recycled (PCR) streams, by comparison, are collected after consumer use and are therefore more heterogeneous, containing impurities, additives, and mixed resins. These complexities reduce overall performance, but crucially, PCR represents the most impactful contribution to circularity, as it enables the reintegration of materials after real-world service life [12,14]. European policies are reinforcing this shift. The forthcoming Regulation on vehicle circularity, intended to replace the current ELV Directive, introduces binding targets for the use of recycled content, requiring up to 25% of plastics in new vehicles to be recycled, with a share directly derived from ELVs through closed-loop schemes [15]. In this context, the development and adoption of thermoplastic elastomers (TPEs) is an excellent alternative to address this challenge. TPEs offer a unique combination of rubber-like elasticity and thermoplastic processability, enabling both performance and recyclability. These materials can be molded, extruded, and thermoformed using standard polymer processing techniques and re-melted for reuse, providing clear advantages over conventional rubbers in a circular economy context. Their dual-phase morphology, comprising soft elastomeric segments and hard thermoplastic domains, confers them with mechanical resilience, good thermal behavior, and the potential to maintain properties across multiple life cycles [16,17].

Among the various classes of TPEs, styrenic thermoplastic elastomer (TPS), particularly those based on block copolymers such as SEBS (styrene-ethylene-butylene-styrene) and SEPS (styrene-ethylene-propylene-styrene), are extensively used in automotive and industrial applications. TPS materials are typically produced by hydrogenating SBS (styrene-butadiene-styrene) copolymers, which significantly improves thermal and oxidative stability. These block copolymers are often blended with polyolefins, commonly polypropylene (PP), to fine-tune mechanical properties and processing behavior [18,19].

In this structure, the polystyrene phase occupies a relatively small volume and exists as discrete spherical domains. These domains are located at the ends of flexible elastomeric chains and serve as multifunctional junction points, functioning similarly to cross-links found in conventional vulcanized rubbers. However, unlike the permanent chemical cross-links in vulcanizates, these connections are physical in nature, making them significantly less stable. At room temperature, the block copolymer exhibited behavior similar to that of vulcanized rubber. Upon heating, the polystyrene domains soften, weakening the physical network and enabling the material to flow. When cooled, the domains resolidify, restoring the original elastomeric properties [20].

An additional important advantage of TPS is that their formulations are composed of constituents, such as polyolefin matrices (e.g., PP), fillers, and lubricants, that are already integrated into established recycling streams [13,21,22,23,24]. This inherent compatibility makes the incorporation of recycled polymers into TPS blends a particularly promising route to reduce environmental impact while maintaining high material performance [25]. The success of such strategies, however, relies on achieving uniform dispersion of the elastomeric phase, as well as preserving key mechanical properties (e.g., tensile strength, elongation at break) and thermal stability [26].

This study aims to evaluate the mechanical and thermal performance of TPS-based elastomeric compounds containing recycled content, in comparison with their virgin counterparts. The novelty of this research resides in the assessment of a TPS blend formulated with recycled components, which contributes to increasing the overall proportion of recycled materials in automotive applications. This investigation is of fundamental importance for understanding the behavior of these next-generation materials in the automotive sector, with the objective of promoting their broader adoption to enhance sustainability, while maintaining essential requirements such as performance, resistance, and aesthetic quality.

## 2. Materials and Methods

### 2.1. Materials

Four commercial styrenic thermoplastic elastomer (TPS) compounds were analyzed in this study. All materials are based on styrene–ethylene–propylene–styrene (SEPS) block copolymers and are specifically formulated for injection molding applications. These compounds are complex, multiphase systems consisting of a soft SEPS elastomeric phase blended with a polypropylene (PP) matrix, a mineral oil-based plasticizer, and various fillers including inorganic particulates and carbon black. The formulations are representative of industrial-grade SEPS-based TPEs typically used in the automotive sector, particularly for exterior applications such as glass encapsulation and windshield sealing systems.

The analyzed materials include two virgin compounds (V-TPS-50 and V-TPS-60) and their recycled-content counterparts (R-TPS-50 and R-TPS-60). The R-TPS-50 and R-TPS-60 compounds contain post-industrial recycled (PIR) and post-consumer recycled (PCR). The virgin compounds serve as reference materials for evaluating the influence of recycled content on performance. More details and the specific proportions of recycled constituents for each formulation are detailed in Table 1**.** The detailed formulation of the samples cannot be disclosed due to industrial confidentiality; however, their general architecture and processing behavior align with those of commercially available counterparts.

### 2.2. Thermal Analysis

Thermogravimetric analysis (TGA) was performed using a TGA Q500 instrument (TA Instruments, New Castle, DE, USA). The measurements were conducted under a nitrogen atmosphere (60 mL/min flow rate), heating the samples from 50 °C to 700 °C at a constant rate of 10 °C/min to evaluate the thermal stability and the different stages of mass loss. To estimate the percentage of inorganic filler and carbon black, the samples were heated from 700 °C to 850 °C, at the same rate, and switched from N_2_ to air at 750 °C (40 mL/min flow rate). The measurements were conducted on an alumina pan, and the weight of each sample was around 12 mg. The calcium carbonate content was estimated by stoichiometric calculation, multiplying the percentage of weight loss observed between 600 °C and 750 °C by the ratio of the molecular weight of CaCO_3_ (100.09 g mol^−1^) to that of CO_2_ (44.01 g mol^−1^).

Differential scanning calorimetry (DSC) analyses were carried out using a DSC Q200 instrument (TA Instruments, New Castle, DE, USA). Samples of approximately 6 mg were sealed in hermetic aluminum pans and tested under a nitrogen atmosphere with a flow rate of 50 mL/min. The thermal analysis consisted of two heating cycles and one cooling cycle, each separated by a 5 min isothermal hold at the end of the respective cycle. The temperature range was from −80 °C to 200 °C, with a constant heating and cooling rate of 10 °C/min.

### 2.3. Soxhlet Extraction

Soxhlet extraction was performed on approximately 10 g of each compound, which was placed in a cellulose thimble and extracted with acetone at 56 °C for 16 h using a Soxhlet apparatus. After extraction, the solvent was removed using a rotary evaporator. The procedure followed was based on ASTM D297-93 [27].

### 2.4. Fourier Transform Infrared (FT-IR) Spectroscopy Analysis

Fourier transform infrared (FTIR) spectroscopy was used to investigate the chemical structures of the samples before and after the extraction process. The tests were carried out with a Spectrum 100 (Perkin Elmer, Waltham, MA, USA) via the attenuated total reflection (ATR) technique with an internal diamond reflection element. The spectra were measured in the wavelength range of 4000–650 cm^−1^ with a resolution of 4 cm^−1,^ collecting 16 scans for each measurement.

### 2.5. Compression Set

Compression set tests were performed in accordance with ISO 815-1-Method A, type B. [28] A constant deformation of 25% was applied to the specimens, which was achieved by introducing specific spacers between the compression plates. To minimize friction between the samples and the compression fixtures, a lubricant is required; in this case, industrial-grade talc was used. The tests were carried out using an Elastocon compression set rig, model EV 03, and all samples were conditioned in an Elastocon air-circulating oven, model EB 19 (Brämhult, Sweden), at two different temperature-time combinations: 70 °C for 22 h and 100 °C for 70 h. Immediately after removal from the oven, the specimens were released and left to recover at room temperature for 30 min before measurement. Residual thickness was measured using a digital thickness gauge, Mitutoyo (Kawasaki, Japan), ensuring high precision in dimensional evaluation. For each material and test condition, three replicate measurements were performed to ensure the reliability of the results. The compression set, expressed as a percentage of residual deformation, was calculated according to the following Equation (1):

(h_0_ − h_l1_)/(h_0_ − h_s_) × 100 (1)

where h_0_ is the initial height, h_l_ the final height, and h_s_ the spacer height.

### 2.6. Mechanical Analysis

Shore A and µ-IRHD hardness were measured using a GiBiTre durometer (GiBiTre s.r.l., Bergamo, Italy) according to ISO 48-4 and ISO 48-2-method M, respectively [29,30]. The tensile properties of samples were measured using the testing machine Zwick-Roell Z020 with a video extensometer videoXtens (ZwickRoell GmbH & Co. KG, Ulm, Germany). Standard specimens were made from the injection-molded plates according to the international standard ISO 37 Type 1 [31]. The load cell employed had a capacity of 500 N, and the displacement velocity of the applied clamp was 500 mm/min. Five test samples were tested at 23 ± 3 °C with 55% of humidity. Tear strength was measured using the same equipment as for the tensile tests, following ISO 34-1 Method A and ISO 6133 for multi-peak trace analysis [32,33]. Due to the limited amount of available material, five replicates were tested for each analysis. Results are reported as mean values ± standard deviation. This approach was used to provide a direct comparison between virgin and recycled formulations while accounting for the experimental variability of the measurements.

### 2.7. Aging Tests

In this study, the mechanical characterization of TPE compounds was performed before and after thermal aging in air to assess their heat resistance over time. This evaluation is crucial, as TPEs can undergo property changes when exposed to elevated temperatures. Accelerated aging simulates long-term thermal exposure in a shorter timeframe, allowing comparison of material performance before and after aging. The thermal aging was conducted at 125 °C for 168 h in an Elastocon EB 19 air-circulating oven (Elastocon AB, Borås, Sweden), following ISO 188-Method B, type 1 [34]. An additional aging test was employed to replicate the effects of solar aging induced by ultraviolet (UV) radiation using a Q-SUN Xe-2 test chamber (Q-Lab Corporation, Westlake, OH, USA). The chamber’s irradiation system features a xenon arc lamp with quartz borosilicate filters, calibrated to deliver an irradiance of 0.55 W/m^2^ at a wavelength of 340 nm. Since the materials under investigation are intended for exterior automotive applications, they were exposed to four levels of accumulated radiant energy, as requested by Stellantis internal procedures: 840 kJ/m^2^, 1260 kJ/m^2^, 2100 kJ/m^2^, and 2520 kJ/m^2^. The test was made in accordance with SAEJ 2527 [35].

### 2.8. Evaluation of Color Change

To quantify color variation, the color coordinates (L*, a*, b*) were measured in the CIELAB color space, and the total color difference ΔE*_ab_ (hereafter referred to as ΔE) was calculated according to the CIELAB 1976 definition using a Konica Minolta CM-3600A (Konica Minolta, Inc., Tokyo, Japan) spectrophotometer [36].

## 3. Results and Discussion

### 3.1. TGA Analysis

**Figure 1:**
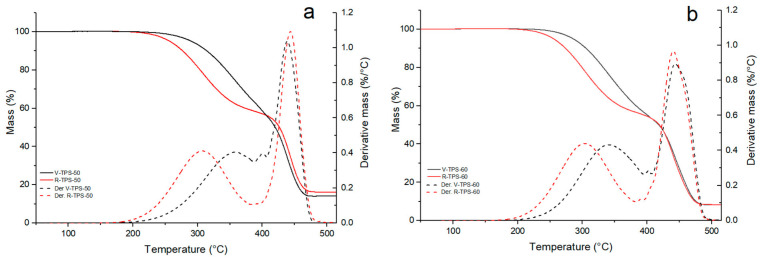
(**a**) TG and DTG of V-TPS-50 and R-TPS-50; (**b**) TG and DTG of V-TPS-60 and R-TPS-60.

To assess and compare the thermal stability of the samples, thermogravimetric analysis (TGA) was performed. TGA curves for this type of commercial material, like rubber compounds, exhibit multiple degradation steps depending on the components present in the blend [37]. The TG and DTG curves for the 50 and 60 series samples in the temperature range of 50 °C to 510 °C are shown in Figure 1a,b. The figure reports the portion of the thermogram most relevant to this discussion, as it highlights the temperature range where the main differences among the samples are observed. The first weight loss, occurring between 200 °C and 300 °C, is attributed to the volatilization of low-boiling-point plasticizing oils. The second weight loss, observed between 350 °C and 500 °C, is related to the degradation of the polymer backbone. Between 600 °C and 750 °C, a further weight loss is associated with the thermal decomposition of calcium carbonate into CO_2_ and CaO [38]. Upon switching to an oxidative atmosphere at 750 °C, an additional weight loss is detected, which is ascribed to the oxidation of carbonaceous fillers, like carbon black (CB). The residual mass at 850 °C is attributed to the presence of inorganic fillers. Thermogravimetric data are reported in the Supplementary Material(Table S1), and the composition results as a function of the weight loss percentage for all samples are reported in Table 2. A comparison of the different samples reveals that the component content in both the 50-series and 60-series materials does not show significant differences between virgin and recycled samples.

When comparing the thermograms of virgin materials with their green counterparts, the onset temperature of oil volatilization is lower in R-TPS-50 and R-TPS-60 than in the corresponding virgin samples. This difference may be due to a variation in the oil composition, which lowers the onset temperature and alters the thermal stability of the compounds [39]. Shi et al. demonstrated that the oil composition significantly affects the flow behavior, thermal stability, and microstructure of SEBS/PP/oil blends. In their study, the naphthenic content, calculated together with the paraffinic and aromatic fractions and inherently present in the lubricating oil of their samples, was found to drive oil migration from the EB/PP phase to the PS phase, plasticizing it and reducing its degradation temperature. This improves processability but decreases thermal stability and promotes oil migration [40].

### 3.2. TGA Investigation After Soxhlet Extraction

**Figure 2:**
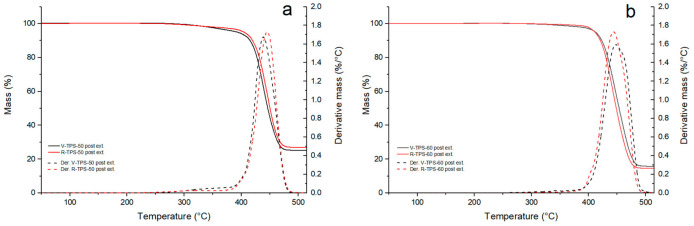
(**a**) TG and DTG of V-TPS-50 and R-TPS-50 after Soxhlet extraction. (**b**) TG and DTG of V-TPS-60 and R-TPS-60 after Soxhlet extraction.

To further investigate the interaction between the plasticizing oil and the polymer matrix in the samples, a Soxhlet extraction was performed using acetone. This was performed to remove extractable compounds, primarily the oil, after which Thermogravimetric Analysis was performed on the residual samples at 10 °C/min up to 600 °C to verify that the oil had been almost completely removed. The thermograms of the extracted samples are shown in Figure 2a,b. After extraction, the volatile content (i.e., the mass loss attributable to volatile/extractable components) was measured by TGA and found to be 4.2% for V-TPS-50, 3.5% for R-TPS-50, 2.6% for V-TPS-60, and 1.6% for R-TPS-60. The higher extractable content in the green materials suggests that in samples with a greater proportion of recycled content, the plasticizing oil exhibits weaker interactions with the polymer matrix compared to virgin materials. This could mean that the oil is less bound or has a low molar mass in the recycled blend, possibly residing more at interfaces or near the surface, which makes it easier to extract [40,41,42].

### 3.3. DSC Analysis

Table 3 summarizes the melting and crystallization parameters obtained from DSC analysis. All samples exhibit an endothermic melting peak around 153 °C and an exothermic crystallization peak between 98 °C and 104 °C, confirming the presence of a semicrystalline polypropylene phase within the investigated TPS compounds [43]. Compared with typical neat PP, which generally shows higher melting and crystallization temperatures, the observed transitions fall within the expected range (120–177 °C for Tm) for multiphase PP/TPE blends containing recycled components [44,45]. The lower melting and crystallization temperatures observed for these materials compared with neat PP may be related to the multicomponent nature of the formulations. In particular, the presence of processing oil/plasticizer and elastomeric phases can interfere with PP chain packing and crystal growth, leading to less regular crystalline structures and a partial reduction in crystallization temperature. This interpretation is consistent with Tomacheski’s findings, who reported a marked reduction in melting and crystallization temperatures when PP was blended with oil and SEBS under different processing conditions, confirming that such effects are directly linked to disrupted crystallinity [46]. Within the present materials, the comparison between virgin and recycled formulations in the 50 series reveals only minor variations in melting or crystallization temperatures, suggesting that the incorporation of recycled material does not markedly alter the crystalline organization of the PP phase. Conversely, in the 60 series, R-TPS-60 exhibits a slightly lower Tc than its virgin counterpart. This difference may be associated with formulation-related effects influencing PP crystallization, such as variations in the oil fraction, elastomeric phase contribution, or recycled components. However, in the absence of direct morphological or compositional evidence, no definitive conclusion can be drawn regarding phase dispersion or compatibilization effects. Overall, the DSC results indicate that recycled content may slightly affect the crystallization behavior of the blends, but without causing major changes in the PP melting transition or compromising the overall thermal stability of the investigated TPS compounds.

### 3.4. IR-ATR

**Figure 3:**
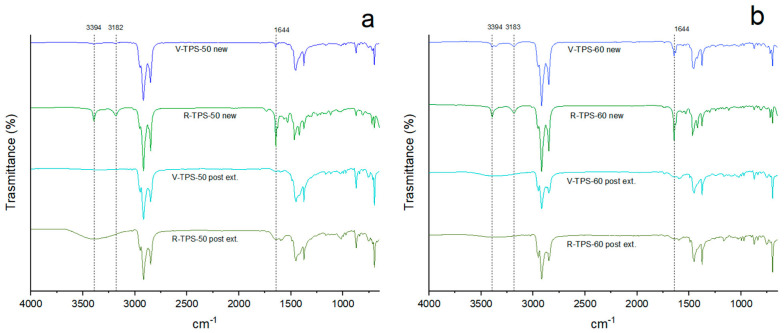
ATR-FTIR spectra of: (**a**) V-TPS-50 and R-TPS-50 before and after Soxhlet extraction, (**b**) V-TPS-60 and R-TPS-60 before and after Soxhlet extraction.

ATR-FTIR spectra of all grades before and after Soxhlet extraction are presented in Figure 3. All samples, virgin and recycled, show characteristic absorption bands of the SEPS-PP polymeric matrix [43]. Specifically, bands at 2950 cm^−1^ and 2850 cm^−1^ correspond to the asymmetric stretching vibrations of CH_3_, while the band at 2918 cm^−1^ is attributed to the asymmetric stretching of CH_2_ [47,48]. Additional bands at 1457 cm^−1^ and 1377 cm^−1^ are assigned to the asymmetric and symmetric bending vibrations of CH_3_ groups, respectively [44,49,50]. A signal at 697 cm^−1^, associated with out-of-plane C–H bending of the aromatic ring in SEPS, is also present [51]. Beyond the typical SEPS-PP spectral features, additional bands are observed at 3394 cm^−1^, 3182 cm^−1^, and 1644 cm^−1^, corresponding to bending, asymmetrical stretching, and symmetrical stretching modes of amide groups, respectively. These features are attributed to fatty acid amides, commonly employed as slip agents such as oleamide and erucamide [52]. Erucamide is typically favored over oleamide in polypropylene-based applications due to its higher thermal resistance. During processing, slip agents migrate toward the surface of the polymer matrix upon cooling, a phenomenon referred to as “blooming.” These result in the formation of a thin, waxy layer that decreases the coefficient of friction, facilitates demolding, and improves scratch resistance [53]. Noh et al. investigated the influence of processing conditions and additive behavior on the scratch resistance of thermoplastic olefins for automotive applications, demonstrating that surface migration of slip agents enhances performance, though the effect may reduce at elevated temperatures [54]. Semi-comparative analysis of virgin and recycled materials seems to reveal a higher intensity of the slip agent-associated bands in the R-TPS-50 and R-TPS-60 samples, suggesting greater surface accumulation due to additive migration [55]. Post-extraction, the recovered oil was identified as paraffinic across all samples analyzed; all the FTIR spectra are in the Supplementary Material (Figure S1) [56]. Residual materials were re-examined by FTIR, and the bands at 3394, 3182 cm^−1^, and 1644 cm^−1^ were no longer significant, indicating the effective removal of both slip agents and mineral oil. No substantial spectral differences were observed between virgin and recycled material pairs following extraction.

### 3.5. Compression Set

**Figure 4:**
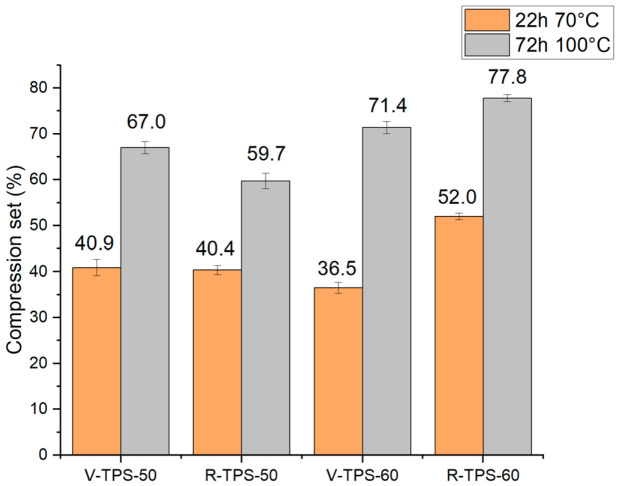
Compression set results.

To evaluate the residual permanent deformation of the materials in this study, compression set tests were performed, and the results are reported in Figure 4. In the 50-series, both V-TPS-50 and R-TPS-50 formulations exhibited similar performance at lower temperatures, with moderate compression set values indicating partial elastic recovery. Moreover, at higher temperatures and extended duration, R-TPS-50 retained better recovery, while the virgin material exhibited higher permanent deformation. In the 60-series, under the less severe testing conditions, the virgin formulation exhibited superior performance compared to the recycled material. However, as test severity increased, by raising both temperature and duration, the performance gap between the virgin and recycled samples decreased. Under these conditions, both materials showed poor recovery, with permanent deformation being particularly pronounced for R-TPS-60. These results indicate that the compression set behavior is strongly formulation-dependent and cannot be attributed to recycled content alone. The TPS-50 and TPS-60 series are industrial multicomponent compounds with different formulation balances and should not be considered as a simple monotonic series differing only in hardness. At 100 °C, elastic recovery depends on the ability of the material to maintain its reversible deformation capacity and to limit irreversible deformation under combined thermal and mechanical loading. This response is influenced by the combined contribution of the elastomeric phase, polyolefin phase, plasticizer fraction, and inorganic components.

The better recovery observed for R-TPS-50 suggests a favorable balance between elastic recovery and resistance to viscous flow, whereas the high permanent deformation of R-TPS-60 indicates reduced thermomechanical stability under compression. Therefore, the deterioration observed in some recycled formulations should be interpreted as the combined effect of composition, plasticizer-related behavior, and thermomechanical response under compression. This interpretation is consistent with the TGA results, which revealed compositional differences between virgin and recycled compounds.

### 3.6. Mechanical Analysis

The mechanical characterization of the TPS compounds began with hardness evaluation, using both Shore A and µ-IRHD scales. The results of the tests are presented in Table 4. In the 50-series, virgin materials exhibited slightly higher hardness than their recycled counterparts, suggesting a mild softening effect, possibly due to lower molecular-weight fractions in the recycled blends. The thermal aging did not significantly change the hardness values of the samples for both hardness scales. In the 50-series, virgin materials exhibited slightly higher hardness than their recycled counterparts, suggesting a mild softening effect. This behavior may be associated with formulation differences, including variations in the plasticizer fraction and its interaction with the polymer phases. Thermal aging did not significantly affect the hardness values in all samples and for both hardness scales. In contrast, the 60-series showed a more complex behavior, with the recycled sample displaying the highest hardness values overall. This could be attributed to a higher degree of structural rigidity. Thermal aging in air induced minimal variations in both hardness scales, indicating a good surface-level structural stability under the applied conditions.

**Figure 5:**
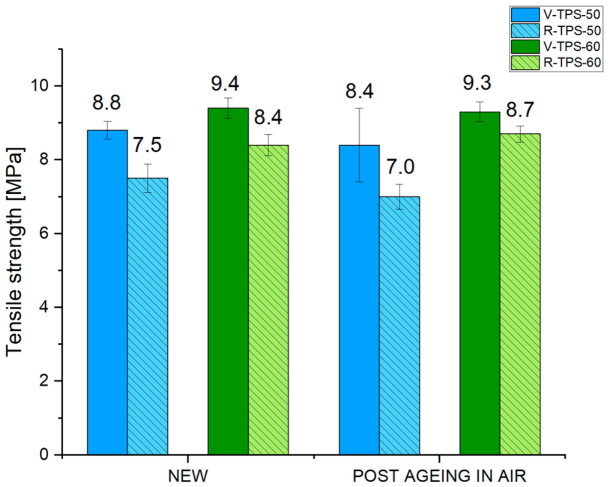
Tensile strength results before and after aging.

The tensile performance of four TPS formulations was assessed in both the as-produced state and after thermal aging in air (Figure 5). In the 50-series, the compounds initially exhibited superior mechanical strength. Upon air aging, both materials experienced a reduction in tensile properties, with tensile strength retention values of 95.5% for V-TPS-50 and 93.3% for R-TPS-50. The slightly lower retention observed for the recycled-content formulation suggests a somewhat greater sensitivity to thermal-oxidative aging, although the overall decrease remained limited. In contrast, the 60-series materials displayed different behavior. While the virgin compound started with the highest tensile strength, it showed a lower retention after aging, equal to 98.9%. Conversely, the recycled formulation R-TPS-60 exhibited a retention value of 103.6%, indicating that the tensile strength was fully retained and even slightly increased after aging. This suggests that in the 60-series, the recycled content does not adversely affect the resistance to thermal-oxidative degradation.

**Figure 6:**
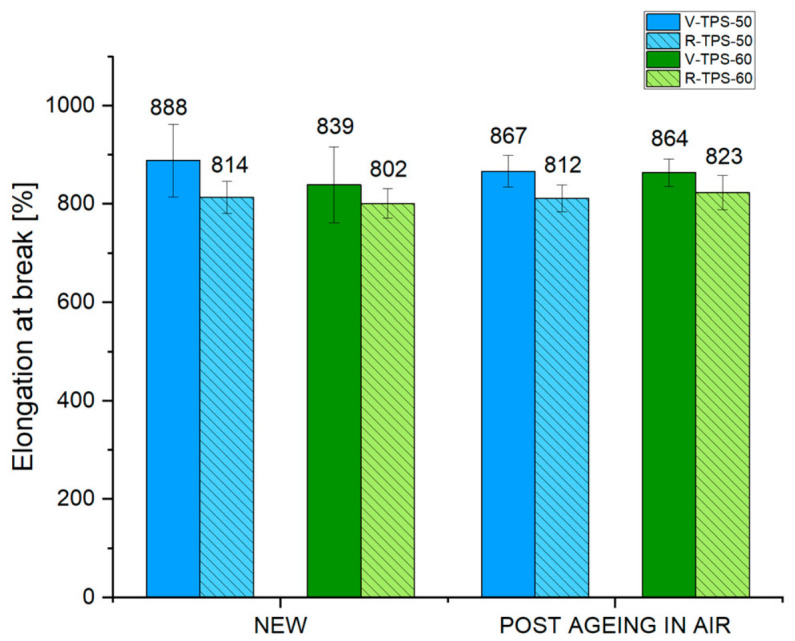
Elongation at break results before and after aging.

The same trend was generally observed in the elongation at break data, which provides insight into the ductility and flexibility of the materials (Figure 6). In the 50-series, the virgin formulation exhibited higher initial elongation than the recycled counterpart. After aging, both samples showed only a limited reduction, with a retention value of 98% for V-TPS-50 and 100% for R-TPS-50. These results indicate that the ductile behavior of both formulations was largely preserved, with the recycled content compound showing an almost complete retention of elongation at break.

In the 60-series, both virgin and recycled formulations maintained high elongation values after aging, with a retention value of 103% for V-TPS-60 and 103% for R-TPS-60. These values slightly above 100% indicate that elongation at break was essentially preserved and marginally increased after aging, which may be associated with experimental variability and thermally induced relaxation phenomena. Overall, the results suggest stable ductile behavior after thermal-oxidative aging for 60-series materials.

**Figure 7:**
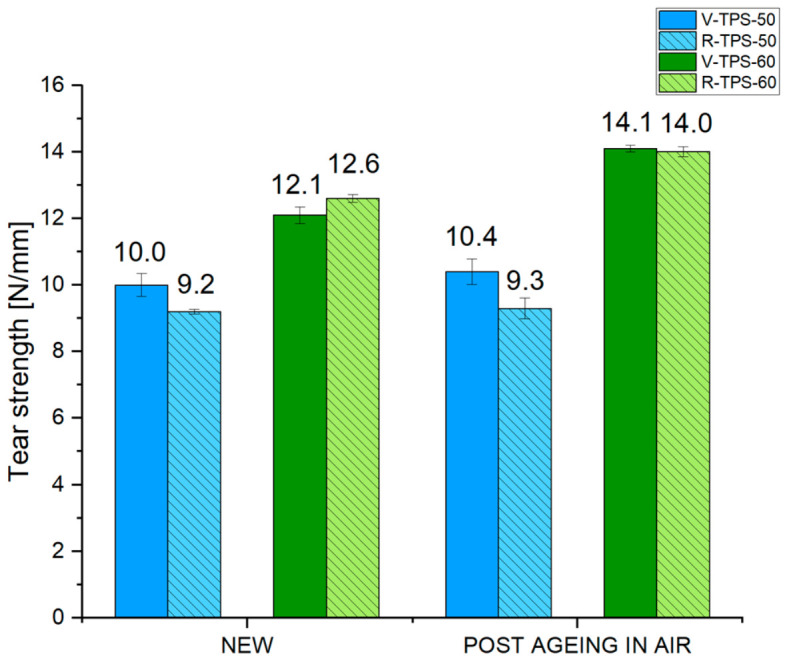
Tear strength results before and after aging.

Tear strength measurements further confirmed these patterns (Figure 7). In the 50-series, both virgin and recycled materials showed low initial tear resistance with only slight increases after aging, corresponding to retention values of 104% for V-TPS-50 and 101% for R-TPS-50. Conversely, the 60-series materials started with higher tear strength and exhibited a more pronounced increase after aging, with retention values of 117% for V-TPS-60 and 111% for R-TPS-60, reaching similar final values. This indicates that the tear resistance of the 60-series materials, regardless of recycled content, was effectively preserved and slightly enhanced after thermal aging. Overall, the mechanical performance of the recycled TPS compounds is consistently comparable to that of their virgin counterparts, with only moderate reductions observed in tensile strength and elongation at break. Tear resistance and hardness values, particularly in the TPS-60 series, demonstrate that the incorporation of recycled content does not necessarily compromise, and can in some cases enhance, specific mechanical characteristics. These findings support the feasibility of using recycled TPS in applications where mechanical integrity is essential, especially when formulations are optimized for higher performance grades.

### 3.7. Color Change Evaluation After UV Aging

**Figure 8:**
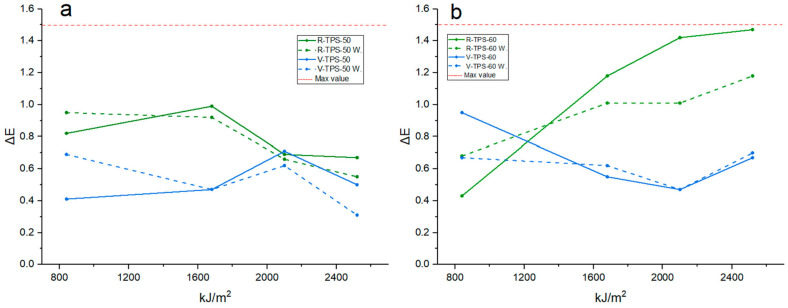
(**a**) ΔE values for V-TPS-50 and R-TPS-50 before and after washing, (**b**) ΔE values for V-TPS-60 and R-TPS-60 before and after washing.

**Figure 9:**
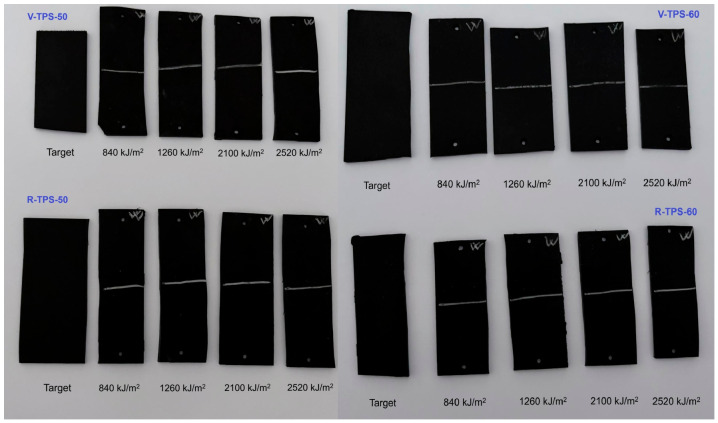
Photographs of the target specimens and of the aged specimens at each aging step. For each sample, the upper half above the white line corresponds to the washed surface, while the lower half corresponds to the non-washed surface for each sample.

The ΔE values for all samples, before and after washing, are shown in Figure 8a,b. In the graph, the blue lines refer to virgin materials, while the green lines represent recycled materials. Solid lines indicate ΔE values measured before washing, whereas dashed lines show the corresponding values after washing with soap and water. The red dashed line at ΔE = 1.5 represents the threshold selected in this study as the limit above which color variation becomes perceptible to the human eye. Representative photographs of the aged specimens are also reported in Figure 9 to provide a visual comparison of the samples after exposure. Since these materials are intended for automotive applications, where surface appearance plays a crucial role in perceived quality and durability, this test is particularly relevant. Evaluating color variation after aging and subsequent cleaning provides valuable insight into the aesthetic stability of the materials under automotive-relevant aging conditions, reflecting industrial validation practice for vehicle applications. The results for 50-series materials indicate that the recycled-content material tends to exhibit slightly higher ΔE values compared to the virgin material, implying greater chromatic sensitivity to aging. Nevertheless, in both cases, the values remain well below the visual perception threshold (ΔE < 1.5), indicating that the observed color changes are minimal. Washing with soap and water caused a slight reduction in ΔE values, which may indicate the presence of temporary surface exudates removed by the treatment. Although the recycled material shows slightly greater color variation than the virgin one, both materials demonstrate good color stability under light exposure. The results for 60-series materials reveal a different behavior between the virgin and green materials. R-TPS-60 shows a progressive increase in ΔE with increasing irradiation, starting from low initial values and approaching the established maximum threshold at the highest exposure level. This trend suggests reduced color stability, particularly in the advanced stages of aging. However, the visual comparison of the aged specimens shows that these variations remain limited and are not clearly appreciable to the naked eye. Surface washing leads to a slight decrease in ΔE values for the recycled material, but this is not enough to bring the values comparable with those of the virgin material. In contrast, V-TPS-60 displays consistent chromatic stability throughout the aging process. Once more, washing results in only minimal changes, confirming the material’s resistance to discoloration and the limited influence of surface residues. From the comparison, it is evident that the virgin material provides a more stable chromatic performance, whereas the green material is more subject to color alteration under prolonged light exposure. The higher ΔE values observed in the recycled-content materials across both sample series are likely due to increased wax migration. The increased surface blooming, as confirmed by FTIR and TGA analyses, suggests a more significant exudation of waxes and oils in these materials. Nevertheless, the ΔE values of the recycled samples remain within acceptable limits, confirming their suitability for applications tolerant to minor color variations.

## 4. Conclusions

This study demonstrates that styrenic thermoplastic elastomer blends containing recycled content can achieve mechanical and aesthetic performances comparable to those of virgin materials, confirming their suitability for demanding applications such as automotive components. Thermal and spectroscopic analyses revealed clear differences between virgin and recycled samples, mainly related to the presence and mobility of plasticizing oils and waxes. Such effects may influence the long-term stability of the materials, as enhanced oil migration could potentially affect surface appearance or durability. Nevertheless, mechanical testing demonstrated that the stabilized recycled blends retain excellent tensile and elastic properties, indicating that the formulation effectively compensates for the presence of mobile oil fractions. After artificial weathering, only minor surface alterations and limited oil exudation were occasionally observed, with no significant color variation. The materials maintained a uniform visual appearance, confirming that these effects are well controlled and do not compromise the overall aesthetic quality. TGA, FTIR, and weathering analyses highlight the central role of oil components in differentiating recycled from virgin formulations. However, the stabilized blends exhibit a well-balanced structure–property relationship, ensuring both mechanical strength and visual durability. These findings confirm the technical feasibility of incorporating recycled fractions into thermoplastic elastomers and support their use as sustainable materials for automotive and related applications.

## Figures and Tables

**Table 1:** Summary of sample information.

Sample	Materials	PIR (wt.%)	PCR (wt.%)	Total Recycled Content (wt.%)	Density (g/cm^3^)
1	V-TPS-50	-	-	-	0.97
2	R-TPS-50	26	43	69	0.97
3	V-TPS-60	-	-	-	0.93
4	R-TPS-60	5	45	50	0.92

**Table 2:** TGA composition results.

Materials	%Volatiles [*w*/*w*]	%Polymer [*w*/*w*]	%CaCO_3_ [*w*/*w*]	%CB [*w*/*w*]	%Residue [*w*/*w*]
V-TPS-50	40.5	45.4	15.5	1.3	6.0
R-TPS-50	40.6	43.5	13.4	1.9	8.1
V-TPS-60	45.3	46.8	7.5	1.8	2.8
R-TPS-60	44.2	47.6	7.5	1.6	3.3

**Table 3:** Average values of the melting peak temperature (Tm) and crystallization peak temperature (Tc) as determined from DSC measurements.

Samples	Tm (°C)	Tc (°C)
V-TPS-50	154	102
R-TPS-50	151	105
V-TPS-60	154	104
R-TPS-60	154	98

**Table 4:** Hardness Shore A and µ-IRHD of all materials before and after aging.

	Shore A		µ-IRHD	
Materials	New	Post Aging	New	Post Aging
V-TPS-50	59.3 ± 1.1	58.8 ± 1.1	68.7 ± 0.6	68.4 ± 0.2
R-TPS-50	55.8 ± 0.5	55.4 ± 1.0	66.9 ± 1.3	64.1 ± 0.9
V-TPS-60	61.6 ± 1.3	63.0 ± 0.4	73.4 ± 0.3	70.2 ± 0.9
R-TPS-60	63.5 ± 0.2	60.2 ± 0.7	75.8 ± 0.9	75.3 ± 0.5

## Data Availability

All data supporting the findings of this study are available within the paper and its Supplementary Material; further inquiries can be directed to the corresponding authors.

## Supplementary Materials

The following supporting information can be downloaded at: https://www.mdpi.com/article/10.3390/polym18131646/s1, Figure S1: FTIR-ATR spectra of extracted oils; Table S1: TGA analysis results.
