# Supplementary material for: Mechanical and Thermal Characterization of Styrenic Thermoplastic Elastomer Compounds with Recycled Content for Sustainable Automotive Applications
Source: Polymers (Basel). 2026 Jul 2;18(13):1646. doi: 10.3390/polym18131646 (PMC13363934; doi:10.3390/polym18131646)
Supplement: Supplementary file 1 [file polymers-18-01646-s001.zip › polymers-4363467-supplementary.pdf]

Mechanical and thermal characterization of styrenic thermoplastic elastomer compounds with recycled content for sustainable automotive applications

Flavia Cano[1], Matilde Arese[1], Graziano Brocani[2], Silvia Ponti[2], Gabriele Ciaccio[2], Valentina Brunella[1*]

[1] *NIS Interdepartmental Centre, Department of Chemistry, University of Turin, Via P. Giuria 7, 10125 Turin, Italy;*
[2] *Stellantis, C.so Agnelli 220, Turin, 10135, Italy*

**Corresponding author:* valentina.brunella@unito.it https://orcid.org/0000-0002-4848-5647

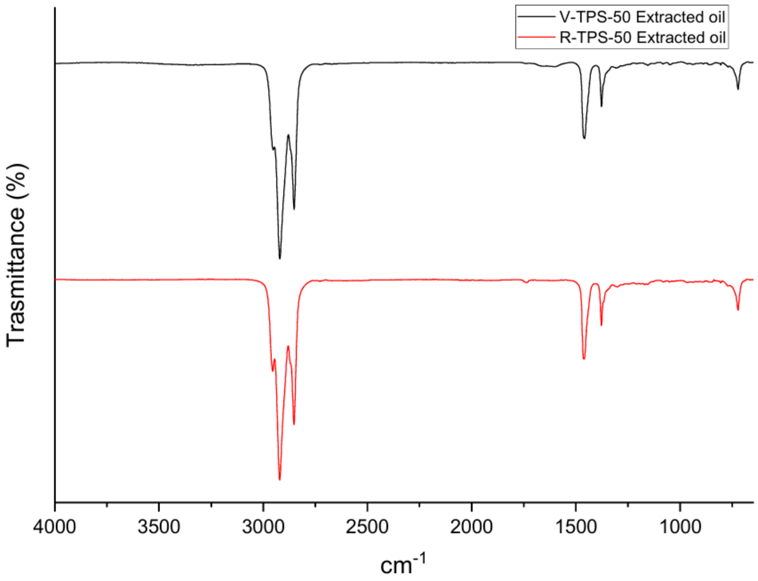

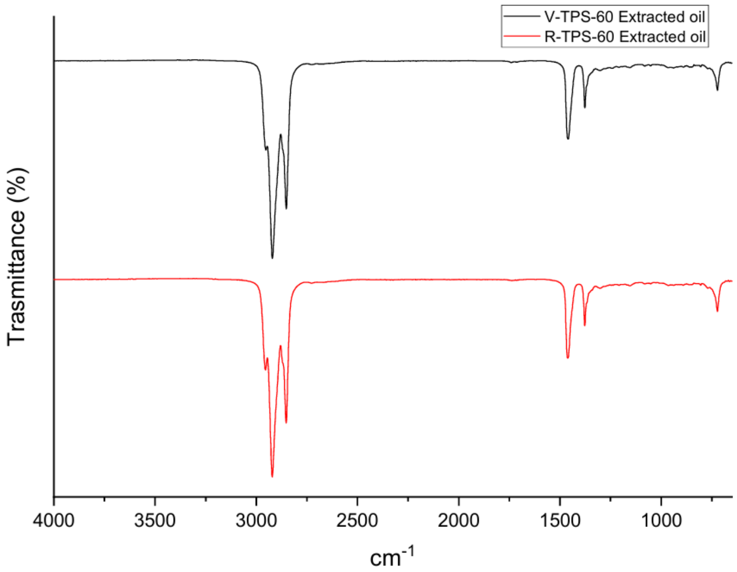

Figure S1: FTIR-ATR spectra of extracted oils

Figure S1 presents the results of FTIR-ATR of the extracted oil from all the TPS materials (V-TPS-50; R-TPS-50; V-TPS-60; R-TPS-60). All spectra have the characteristic peaks of paraffinic oil, the peaks in the 2850–3000 cm^{-1} region corresponds to the symmetrical stretching vibration of $-CH_3$ and $-CH_2$ group in the aliphatic chains. The peaks at around 1370-1450 cm^{-1} belong to $-CH_2$ bending and the peak at 721 cm^{-1} represents the rocking vibration of $-CH_2$.

Table S1: TGA analysis results

Materials	T_{onset1}/°C	T_{onset2}/°C
V-TPS-50	296	424
R-TPS-50	254	426
V-TPS-60	285	427
R-TPS-60	253	422

In **Table S1** the onset temperatures obtained from TGA are reported. T_{onset1}, related to the volatilization/degradation of low-molecular-weight compounds such as oils, shows some variability between virgin and recycled materials, suggesting differences in the volatile fraction. In contrast, T_{onset2}, associated with polymer degradation, remains nearly constant, indicating similar thermal stability of the polymer matrix.
